# Impact of Metabolic States on SARS-CoV-2 Vaccine Responses in Mouse Models of Obesity and Diabetes

**DOI:** 10.3390/covid5010002

**Published:** 2024-12-24

**Authors:** Olivia A. Smith, Brent Fujimoto, Teri Ann S. Wong, Albert To, Troy Odo, Aquena Ball, Brien K. Haun, Hiromi Muramatsu, Ying K Tam, Norbert Pardi, Axel T. Lehrer

**Affiliations:** 1 Department of Tropical Medicine, Medical Microbiology, and Pharmacology, University of Hawaii Manoa, Honolulu, HI 96813, USA; 2 Cell and Molecular Biology Graduate Program, University of Hawaii Manoa, Honolulu, HI 96813, USA; 3 Department of Microbiology, Perelman School of Medicine, University of Pennsylvania, Philadelphia, PA 19104, USA; 4 Acuitas Therapeutics, Vancouver, BC V6T 1Z3, Canada

**Keywords:** SARS-CoV-2 vaccine, obesity, diabetes, mouse model, mRNA vaccine, subunit vaccine, cell-mediated immunity, humoral immunity, antibody avidity

## Abstract

The emergence of SARS-CoV-2 has resulted in a significant impact on public health, particularly for individuals with underlying health conditions such as obesity and diabetes. While vaccination efforts have played a crucial role in reducing hospitalizations, it remains unclear whether the effectiveness of these vaccines varies among different population groups. In this study, we investigated the immune responses generated by various SARS-CoV-2 vaccine platforms in mouse models with obesity and diabetes, focusing on both cell-mediated and humoral immune responses. Our findings revealed diminished immune responses in diabetic and obese mice compared to healthy counterparts. After vaccination with adjuvanted subunit or mRNA lipid nanoparticle (LNP) vaccines, both humoral and cell-mediated responses were significantly reduced in diabetic mice. Obese mice also exhibited decreased immunogenicity, albeit to a lesser extent. However, it should be noted that mRNA vaccines demonstrated strong neutralizing responses across all metabolic states, while adjuvanted subunit vaccines elicited higher antibody avidity in mice with type 2 diabetes (T2D) and obesity compared to healthy mice. These results suggest that the impaired humoral and cell-mediated responses observed in altered metabolic states may be linked to chronic inflammation associated with obesity and suboptimal glycemic control in diabetes. Understanding the impact of these metabolic disturbances on vaccine immunogenicity is crucial for developing optimized vaccines that can effectively enhance immune responses and provide long-lasting protection against SARS-CoV-2, even in individuals with obesity and diabetes. By contributing these findings, we support efforts to improve vaccine efficacy in populations affected by metabolic disorders, advancing effective immunization against SARS-CoV-2.

## 1. Introduction

Coronavirus disease (COVID-19), caused by the betacoronavirus, SARS-CoV-2, led to a global pandemic starting in December 2019 [1]. The pandemic has led to societal and community-level medical deficits, economic disruption, and unprecedented fatalities. Additionally, the pandemic increased health disparities, especially in those with comorbidities [2,3]. Comorbidities, including type 2 diabetes mellitus (T2D), can result in higher hospitalization rates, more severe COVID-19, and increased deaths [4]. Individuals with T2D and obesity comorbidities accounted for a significant portion (41.7%) of COVID-19 hospitalizations in the United States [4]. Over 100 million Americans live with diabetes mellitus (DM) or prediabetes, metabolic disorders characterized by insulin resistance and altered glucose homeostasis [5–7]. Despite this risk, few studies have compared vaccine responses between T2D and healthy individuals [8,9]. Even fewer studies have attempted to find ways to improve vaccines for people with T2D.

At the end of 2020, mRNA vaccines were available through emergency use authorization (EUA) and later approved for use [10]. Additional SARS-CoV-2 vaccine platforms have been developed during the pandemic, including adenoviral vector COVID-19 vaccines, inactivated whole virus SARS-CoV-2 vaccines, and recombinant protein vaccines [11]. Alternative vaccine platforms, including protein subunit vaccines, may increase protection against severe disease in those with altered metabolic states.

While genetic vaccines can be limiting due to pre-existing anti-vector immunity, inactivated whole virus vaccines may require additional safety precautions due to potential incomplete virus inactivation, and attenuated vaccines could lead to reversion of the virulent strain [12]. Further, mRNA vaccines require stringent cold-chain storage methods [13]; however, recombinant protein vaccine candidates remain a vaccine safety and accessibility standard, although they require the isolation, purification, and formulation of specific antigenic components. This process might be more complex and time-consuming compared to mRNA vaccine development [14]; however, recombinant protein vaccines are already mass-produced for other viral diseases, including influenza, making production at commercial scale from various expression systems readily accessible [15].

We compared two vaccine platforms, nucleoside-modified mRNA formulated in lipid nanoparticles (LNPs) and recombinant protein vaccines adjuvanted with either CoVaccine HT or Alum, expected to generate diverse responses using mouse models to demonstrate humoral and cell-mediated immunity in healthy animals and those with altered metabolic states. SARS-CoV-2 anti-spike antibodies (Abs) were analyzed as a presumptive correlate of protection for SARS-CoV-2 infection, as Abs are the more established measure of immunity in all instances. At the same time, cell-mediated immunity was also assessed as a potential additional mechanism involved in protection [16,17].

Due to the complexity of diabetes, induction of mimicked diabetic states in mouse models has previously been a challenge [18]. To address this, three models of diabetes, previously shown to mimic states of obesity and diabetes, were used [18–20]. The chemotherapeutic agent streptozotocin (STZ) induces beta cell death, thereby inducing a type 1 diabetic (T1D)-like state. Additionally, C57BL/6 obese (OB) mice, given a high-fat diet, are used to indicate a prediabetic state in mice. The combination of obesity and the addition of the chemotherapeutic agent STZ leads to a diabetic state most like T2D seen in humans compared to alternative monogenic mouse models of diabetes [21]. These three metabolic states were used as indicators of metabolic change to determine differences in humoral and cellular immune responses within these alterations after receiving doses of vaccines from differing vaccine platforms.

The perturbations in the immune system of humans with obesity and diabetes include elevated T_h_1 and Th17-associated cytokines and depressed T_h_2 cytokines [22,23]. This seems paradoxical because T_h_1 cytokines correlate with a robust antiviral response, yet obesity and DM are associated with poor outcomes for virtually all virus-mediated diseases, particularly COVID-19 [24–26]. An adjuvant consistently stimulating the highest T_h_1/T_h_17 versus T_h_2 responses may initiate robust protection in all metabolic states [27]. However, individuals with obesity and DM show similar overall antibody titers in response to influenza vaccines as healthy individuals yet are still more susceptible to infection [8,9]. Persons with obesity and DM may need higher IgG levels or a more focused immune profile (potentially geared more towards a T_h_1 response).

Recombinant SARS-CoV-2 spike protein vaccines adjuvanted with CoVaccine HT (or alum) induce high levels of virus-neutralizing antibodies against SARS-CoV-2 [28,29]. Furthermore, CoVaccine HT-adjuvanted vaccines produced the highest anti-spike IgG concentrations after only one immunization compared to alum-adjuvanted vaccines [28,29]. IFN-*γ* -secreting T-cells were more abundant in mice receiving the subunit vaccine adjuvanted with CoVaccine HT than alum, suggesting a shift to a more balanced T_h_1/T_h_2 response [29].

An optimal vaccine will increase immunity to SARS-CoV-2, despite an immune system affected by obesity and DM. Metabolic syndromes, including obesity and DM, affect baseline immunity through a chronic inflammatory state, with increased adipokines and cytokine-like hormones [30–32]. Epidemiologically, it has been shown that metabolic syndromes can reduce humoral responses to vaccines, including SARS-CoV-2 [33–36], which may be due to these alterations in basal immunity in both obesity and diabetes; however, this remains unclear.

Additionally, glycemic control may impact responses to vaccines. Previous epidemiological data suggest weakened immune responses to SARS-CoV-2 vaccination in patients with T2D and poor glycemic control compared to T2D patients with healthy glycemic control [37]. The complexity of metabolic syndromes, including obesity and diabetes, increase variabilities associated with the effects of immune responses to vaccination, making identifying a singular event or cause unlikely. The combination of basal immune alterations, glycemic control, and apoptosis of beta cells all likely contribute to the affected humoral response to vaccination.

This novel study analyzes the effects of altered metabolic states on humoral and cell-mediated responses to SARS-CoV-2 vaccination using mouse models of obesity and diabetes and high-throughput assay technology.

## 2. Materials and Methods

### 2.1. Mouse Experiments

#### 2.1.1. Ethical Statement

All mouse experiments were approved by the University of Hawai’i Institutional Animal Care and Use Committee (IACUC) and conducted in strict accordance with local, state, federal, and institutional policies established by the National Institutes of Health and the University of Hawai’i IACUC. The University of Hawai’i, John A. Burns School of Medicine (JABSOM) Laboratory Animal Facility is accredited by the American Association for Accreditation of Laboratory Animal Care (AAALAC). All animal experiments were conducted in consultation with veterinary and animal care staff at the University of Hawai’i.

#### 2.1.2. Animals and STZ Treatment

For mice treated with a high-fat diet, 16-week-old male C57BL/6J mice fed a high-fat diet (Research Diets Inc., New Brunswick, NJ, USA; cat# D12492i; containing 60% kcal fat) starting at six weeks of age were purchased from The Jackson Laboratory (The Jackson Laboratory, Bar Harbor, ME, USA; cat# 380050). Female mice were not used as female mice are less susceptible to diet-induced obesity and diabetes induction. Upon receipt, high-fat-diet mice were maintained on Research Diets Inc D12492i 60% kcal fat diet for the remainder of the study. For mice given a control diet, three-week-old male and female mice were purchased from The Jackson Laboratory (The Jackson Laboratory cat# 000664). Upon receipt, control diet mice were maintained on Teklad global soy protein-free extruded 2020x diet (Envigo, Indianapolis, IN, USA; 16% kcal fat diet) for the entirety of the study.

At 16 weeks of age, selected mice were treated with STZ (Cayman Chemical Company, Ann Arbor, MI, USA; cat# 13104) via an intraperitoneal injection of 40 mg/kg STZ diluted in citrate buffer (0.023M sodium citrate, 0.027 citric acid; pH 4.5) for five consecutive days. STZ was injected within ten minutes following dilution in citrate buffer. Mice were fasted for four hours prior to STZ injection. Dosages were adjusted based on the weight of the individual mice.

### 2.2. Recombinant Protein Expression and Purification

The plasmid was generated to express the pre-fusion, protease-resistant, trimeric transmembrane (TM)-deleted spike (S) glycoprotein from SARS-CoV-2 as described previously [28]. Modifications to the gene include the removal of the furin and S2’ cleavage site, adding two prolines between the heptad repeat one and central helix region, and a foldon trimerization domain. Briefly, *Drosophila* S2 cells were transfected with the S gene using Expifectamine Sf (Invitrogen, Carlsbad, CA, USA) according to the manufacturer’s instructions using ExCell420 media (Sigma-Aldrich, St. Louis, MO, USA). Serial passage of the cell culture into fresh media spiked with 300 μg/mL hygromycin B generated a stably transformed cell line. The cell culture was expanded to 1L in a 2L Cellbag^™^ bioreactor on a WAVE bioreactor system (Cytiva, Marlborough, MA, USA) and induced with 200 μM CuSO_4_ the following day. The culture supernatant was harvested one week later, and sodium azide (Sigma-Aldrich, St. Louis, MO, USA) was added to 0.01% (*w*/*v*) before cold storage or purification. While excessive glycosylation may affect immunogenicity by masking antigenic epitopes, the proteins produced were not excessively glycosylated, therefore not impacting immunogenicity.

Recombinant S protein was purified from clarified cell culture supernatants by immunoaffinity chromatography (IAC) using the SARS-CoV-2 cross-reactive mAb CR3022 (provided by Mapp Biopharmaceutical, RRID:AB_2848080) coupled to NHS-activated Sepharose at a concentration of 10 mg/mL. The antigen was eluted with a glycine buffer (pH 2.5) in tandem into a HiPrep 26/10 desalting column (Cytiva, Marlborough, MA, USA) equilibrated with PBS. The oligomeric content was evaluated by size-exclusion chromatography using a HiLoad 16/600 column (GE Healthcare, Piscataway, NJ, USA), equilibrated with PBS. The S protein eluted as a single peak. The final product migrated as two bands, corresponding to the monomer and trimer on SDS-PAGE, under denaturing conditions and was reactive to CR3022 mAb on a western blot. Antigens were filter-sterilized with a 0.22 μm syringe filter (Cytiva, Marlborough, MA, USA) and stored at −80 °C until use.

### 2.3. mRNA-LNP Vaccine Production

The mRNA vaccine was designed based on the SARS-CoV-2 Spike protein sequence (Wuhan-Hu-1, GenBank: MN908947.3). The coding sequence of the full-length delta-furin S protein (RRAR furin cleavage site abolished between amino acids 682–685) is described in Laczko et al. [38]. The mRNA was produced as described [39]. Briefly, the coding sequence was codon-optimized, synthesized, and cloned into the mRNA production plasmid (GenScript). The mRNA was transcribed to contain a 101-nucleotide long poly(A) tail and m1Ψ−5′-triphosphate instead of uridine-5′-triphosphate was incorporated into the mRNA. Capping of the in vitro transcribed mRNAs was performed co-transcriptionally using the trinucleotide cap1 analog, CleanCap (TriLink). The mRNA was purified by cellulose purification as described [40]. Cellulose-purified m1Ψ-containing RNAs were encapsulated in lipid nanoparticles containing an ethanolic lipid mixture of ionizable cationic lipid, phosphatidylcholine, cholesterol, and polyethylene glycol-lipid at an acidic pH. The mRNA-loaded particles were shipped on dry ice and stored at −80 °C at a 1 mg/mL concentration (from the University of Pennsylvania, Philadelphia, PA, USA).

### 2.4. Immunization (Dosage)

Groups (n = 37) of 6–8-week-old C57BL/6 male mice, bred in-house at the University of Hawai’i, were immunized intramuscularly (i.m.) at days 0 and 21 with 3 μg of SARS-CoV-2 S protein in combination with 1 mg of CoVaccine HT^™^ adjuvant (Protherics Medicines Development Ltd., a BTG company, London, UK) (an oil-in-water emulsion of hydrophobic, negatively charged sucrose fatty acid sulphate esters with the addition of squalane [41,42]), or 9 μg mRNA, 3 μg mRNA, or 1 μg mRNA. The negative control groups received equivalent doses of adjuvant only (CoVaccine HT^™^) or luciferase mRNA [38]. Protein subunit vaccines were prepared by mixing the liquid adjuvant with antigen diluted in 1× PBS to the desired concentration immediately prior to administration to mice. The spike antigen was absorbed to alum by rotation for 1 h at room temperature (RT). The supernatant was decanted after centrifugation, and the alum slurry was resuspended with the same volume of sterile PBS. The mRNA vaccine was freshly thawed before injection.

### 2.5. Immunization (Diabetic Mouse Model Experiment)

Mouse groups (n = 104) of 24-week-old C57BL/6 male mice (Jackson Laboratory) were immunized intramuscularly (i.m.) at days 0 and 21 with 3 μg of SARS-CoV-2 S protein in combination with 1 mg of CoVaccine HT^™^ adjuvant (Protherics Medicines Development Ltd., a BTG company, London, UK), Alhydrogel^®^ adjuvant 2%, “alum” (100 μg per dose) (InvivoGen, San Diego, CA, USA), or mRNA SARS-CoV-2 Spike at 3 μg. Alhydrogel^®^ is a colloid of aluminum hydroxide that binds protein to facilitate antigen uptake [43]. Vaccines were prepared for injection as described above.

### 2.6. Glucose Tolerance Test

For the glucose tolerance test (GTT), overnight-fasted mice were injected intraperitoneally with glucose (1 mg/kg, adjusted individually for mouse weight) diluted in PBS. Blood glucose levels were measured using a OneTouch Ultra handheld glucometer (Lifescan, Malvern, PA, USA). Samples were collected over 2 h at 30 min intervals—blood samples were collected by clipping the distal ~1 mm of the tail. For repeated sampling, the surface of the original wound was disrupted.

### 2.7. Analysis of Antibodies by Multiplex Microsphere Immunoassay (MIA)

A multiplex microsphere-based immunoassay measured the IgG antibody concentration in mouse sera [28,29]. Magnetic MagPlex^®^ microspheres (Luminex Corporation, Austin, TX, USA) were coupled to purified S protein or bovine serum albumin (BSA) as control. A mixture of S- and BSA-coupled beads (approximately 1250 beads each) was incubated with diluted sera in black-sided 96-well plates for 3 h at 37 °C with gentle agitation in the dark. Following two washes with MIA buffer (1% BSA and 0.02% Tween 20 in 1x PBS), 50 μL of 1 μg/mL red phycoerythrin (R-PE)-conjugated goat anti-mouse IgG antibodies (Jackson ImmunoResearch, Inc., West Grove, PA, USA) were added and incubated at 37 °C for another 1 h. After washing with the MIA buffer twice, the beads were resuspended in MAGPIX^®^ drive fluid and analyzed on a MAGPIX^®^ Instrument (Luminex Corporation, Austin, TX, USA). The experimental samples’ median fluorescence intensity (MFI) readouts were converted to antibody concentrations using purified antibody standards prepared from pooled mouse antiserum to S protein. The standard curve of anti-spike IgG was established using IgG concentrations of 4.88–1000 ng/mL and analyzed in the MIA assay. The resulting MFI values were analyzed using a sigmoidal dose–response, variable slope model (GraphPad Prism, San Diego, CA, USA), with antibody concentrations transformed to log10 values. The resulting curves yielded r^2^ values > 0.99 with a well-defined top and bottom and the linear range of the curve was used to determine concentration in the experimental samples. The experimental samples were analyzed side by side with the antibody standards at different dilutions (1:2000, 1:10,000, 1:20,000, 1:80,000, or 1:160,000) to obtain MFI values that fall within the linear range of the standard curve. The same computer program interpolated the experimental sample IgG concentrations from the standard curves. Finally, the interpolated values were multiplied by the dilution factors and plotted as antibody concentrations (ng/mL or μg/mL).

### 2.8. Splenocyte Preparation and ELISpot Assays

Splenocytes were harvested ten days after the second vaccination (day 31), and single-cell suspensions were prepared by mechanically homogenizing each spleen with frosted glass slides in RPMI-1640 medium supplemented with 10% FBS, penicillin (100 units/mL), and streptomycin (100 μg/mL). Red blood cells were lysed using 1× eBioscience^™^ RBC Lysis Buffer ((Invitrogen, Carlsbad, CA, USA), and the cell pellet was resuspended in the kit-supplied CTL-Test media supplemented with 1% L-glutamine. According to the manufacturer’s instructions, the ELISpot assay was performed using the mouse IFN-γ/IL-4/TNF-α/IL-2 ELISpot kit (Cellular Technology Limited, Shaker Heights, OH, USA). A total of 2.5 × 10^5^ cells/well were added to a 96-well PVDF membrane plate pre-coated with IFN-γ/IL-4/TNF-α/IL-2 Capture Solution the previous night. The cells were stimulated in duplicates for 24 h with a peptide pool consisting of 17-mer peptides with ten amino acids overlapping, covering the spike protein of SARS-CoV-2 (BEI Resources NR-52402) at 10 μg/mL or medium containing an equal concentration of DMSO (0.05%) as the negative control. The same concentration of cells was incubated with a cell activation cocktail (BioLegend, San Diego, CA, USA) at 1:500 dilution containing phorbol-12-myristate 13-acetate (PMA, 0.081 mM) and Ionomycin (1.3386 mM) as the positive control or with CTL-Test Media only to determine background activation signal. Plates were developed using specific monoclonal detection antibodies, followed by an HRP- and AP-conjugated secondary antibody. Spots were visualized using the supplied Developer Solution. The reaction was stopped by decanting the solution and rinsing the plate with tap water before air drying. The spots were enumerated using the CTL ImmunoSpot^®^ S6 Universal Analyzer (Cellular Technology Limited, CTL, Shaker Heights, OH, USA), and the number of antigen-specific cytokine-secreting spot-forming cells (SFCs) per million cells for each stimulation condition was calculated by subtracting the number of spots detected in the CTL test medium-only wells. The media-only values were subtracted from the stimulated values, divided by 3 × 10^5,^ and multiplied by 10^6^ to obtain the SFC/10^6^ splenocytes.

### 2.9. Microneutralization Assay

The virus-neutralizing capacity of immune mouse serum was determined in a microneutralization test. The serum dilution yielding 50% virus inhibition, expressed as NT50, was reported as the neutralization titer. VSV-SARS-CoV-2-GFP was obtained from Rocky Mountain Laboratories (RML) (Hamilton, MT, USA) and was cloned using the base vaccine vector [44,45], where GFP was added as an additional open-reading frame [44]. The rVSV-SARS-CoV-2-GFP was grown in Vero E6 cells [46]. One day before the assay, Vero cells were seeded in a 96-well plate at 2.5 × 10^4^ cells per well in DMEM-10 medium (Gibco, Waltham, MA, USA). Serum samples were heat-inactivated at 56 °C for 30 min before use. Six four-fold serial dilutions starting at 1:40 of each serum sample were incubated with rVSV-SARS-CoV-2-GFP at a final concentration of 2 × 10^4^ PFU/mL in DMEM-2 medium (50 μL) for 1 h before being added to Vero cell monolayers in quadruplicate.

Virus-positive cells were visualized and counted 22 h later using the CTL Immunospot S6 (Cellular Technology Limited, Shaker Heights, OH, USA). The NT50 titers were determined by non-linear regression analysis (GraphPad Prism 9.3.1). Sera from mice immunized with PBS + CoVaccine HT from the same time points were used as negative controls. The lowest serum dilution, 1:40, determined the negative cutoff. Negative values are represented as an NT50 of 20 in the figures.

### 2.10. Avidity Assay

Concentrations of anti-spike IgG in mouse sera were standardized, where equivalent concentrations of anti-spike IgG were present for the assay. Fifty microliters of standardized sera were incubated with antigen-coupled microsphere beads for 3 h at 37 °C. Incubation was followed by two rounds of washing with MIA wash buffer. Antibody–bead complexes were incubated with 50 μL of increasing concentrations (0.5 M, 1 M, 2 M, 4 M, or 6 M) ammonium thiocyanate (NH_4_SCN) for 10 min. MIA buffer was added to the control wells not treated with NH_4_SCN. Three rounds of washing were followed by a one-hour incubation with (R-PE)-conjugated goat anti-mouse IgG antibodies. After washing with the MIA buffer twice, the beads were resuspended in MAGPIX^®^ drive fluid and analyzed on a MAGPIX^®^ Instrument. Relative avidity was calculated at the 4 M NH_4_SCN concentration by dividing the MFI of a treated sample by the sample MFI in the untreated condition.

### 2.11. Statistical Methods

Graphs show mean ± standard deviation unless otherwise indicated. Statistical comparisons between the subgroups were assessed by one-way or two-way analysis of variance (ANOVA). In all other cases, a Student’s *t*-test was performed to calculate *p* values and determine if there was a significant difference between the two groups. *p* values of less than 0.05 were considered statistically significant. Area under the curve (positive incremental) values were calculated based on the trapezoid model, relative to the baseline, using the GraphPad Prism software (GraphPad, Inc., San Diego, CA, USA).

## 3. Results

### 3.1. Comparative Humoral Responses to mRNA LNP Vaccines and Adjuvanted Subunit Vaccines at Equivalent Concentrations

To determine the subunit and mRNA dosages that produce similar levels of antibody and cytokine secretion, we compared 3 μg of pre-fusion spike (S) glycoprotein from SARS-CoV-2 adjuvanted with CoVaccine HT [28], with 9, 3, and 1 μg of mRNA LNP vaccine with a comparable amino acid sequence, sans pre-fusion stabilization [38]. The pre-fusion stabilized protein represents the spike (S) protein of SARS-CoV-2 in the native conformation before undergoing conformational change to facilitate viral entry into host cells [29,38].

**Figure 1.**
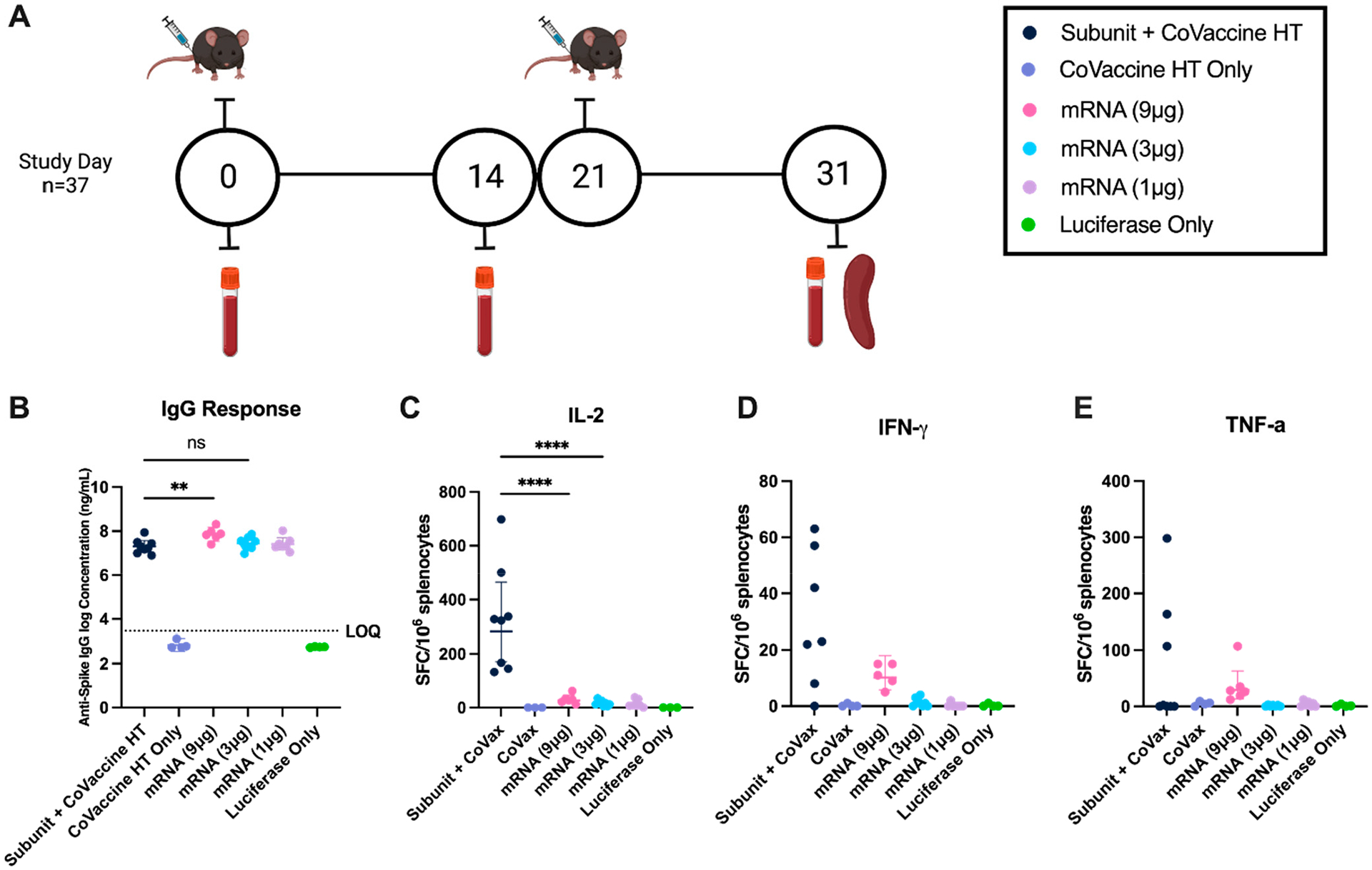
Dose range for mRNA vaccine candidate compared to the subunit vaccine in C57/BL6 mice. (**A**) Vaccine schedule. Serum was collected at baseline, and mice were immunized at days 0 and 21. Two weeks following dose one, serum was collected (day 14). Ten days following the second immunization (day 31), mice were sacrificed for serum and splenocyte collection. (**B**) IgG concentrations of individual animals ten days after the second dose (day 31). The optimal dose was chosen based on achieving a similar magnitude of humoral response to the mRNA vaccine (IgG concentration), not the highest antibody titer. (LOQ: limit of quantification). FluoroSpot (ELISpot) responses of re-stimulated splenocytes after the second dose: IL-2 (**C**), IFN-*γ* (**D**), and TNF-a (**E**). Data were analyzed using a one-way ANOVA with repeated measures, geometric mean with 95% confidence interval (CI) shown. Symbols represent individual animals (**** = *p* < 0.001; ** = *p* < 0.01; ns = no significance).

Healthy C57BL/6 mice were given two intramuscular (IM) doses twenty-one days apart. Ten days after the second dose (day 31), serum and splenocytes were collected (Figure 1A). The collection day was chosen to appropriately assess cell-mediated immunity while accounting for the kinetics of the humoral response. Mice given 3 μg of mRNA vaccine had comparable spike-specific IgG to mice given 3 μg of subunit vaccine (Figure 1B). The 3 μg mRNA dose was chosen as the optimal dose due to its comparable magnitude of humoral response to the subunit vaccine, despite not producing the highest antibody titer. ELISpot analysis demonstrated that 3 μg of subunit vaccine induced more IL-2 (Figure 1C) and IFN-*γ* (Figure 1D) spot-forming cells (SFCs) than 1, 3, or 9 μg of mRNA vaccine. Significant differences in spike protein-stimulated TNF-α-secreting splenocytes were not observed between mice given the different vaccines (Figure 1E), although a trend towards higher cytokine-producing cell counts within the subunit vaccine group was observed.

### 3.2. Diabetic States Are Induced via STZ Treatment and/or Diet-Induced Obesity

**Figure 2.**
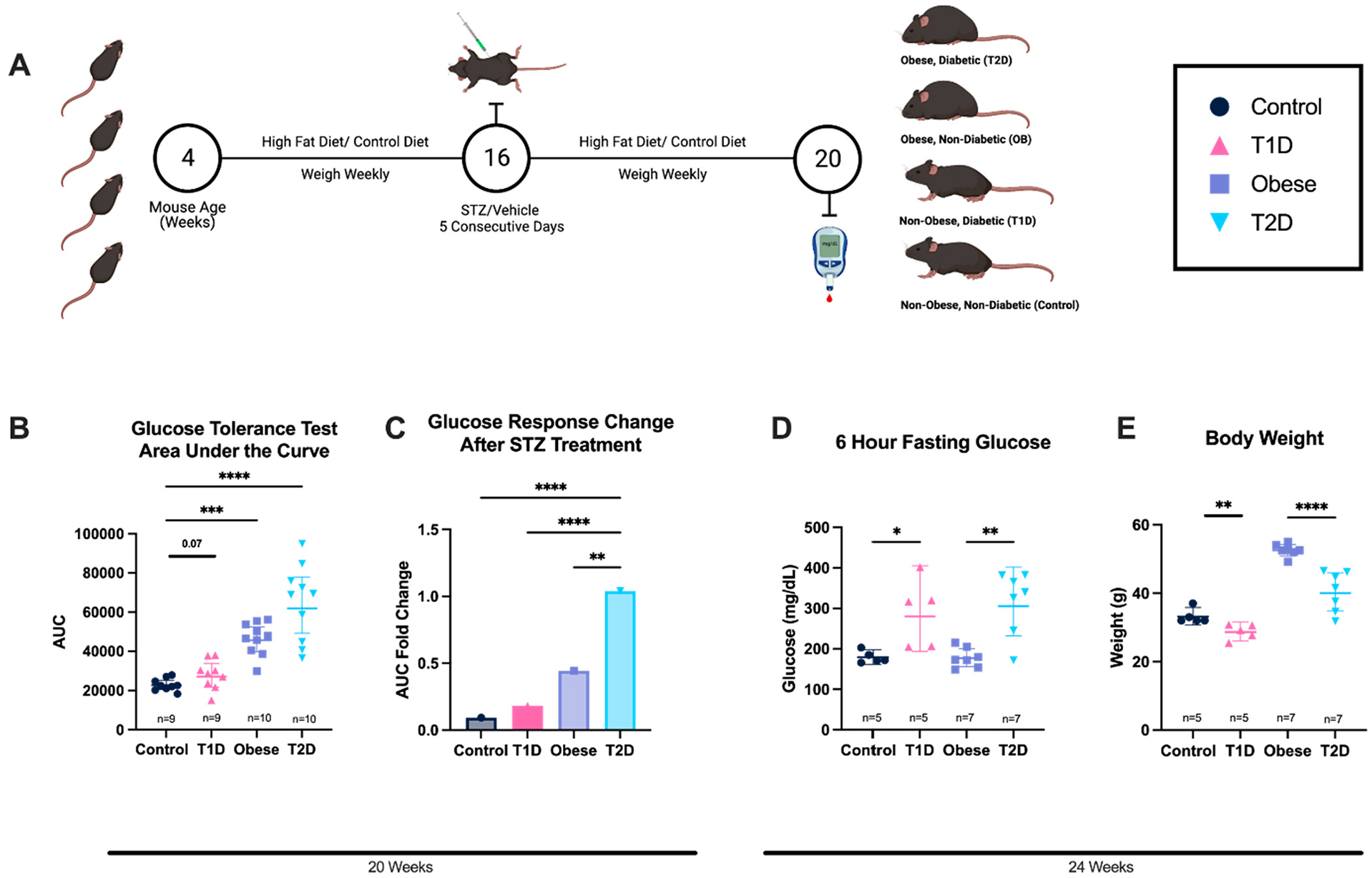
Diabetic states are induced via STZ treatment and diet-induced obesity. (**A**) Graphical representation of diabetes and obesity induction from 4 to 20 weeks of mouse age. Induction of diabetes through STZ injections at 16 weeks of age and confirmation of diabetic status at 20 weeks. (**B**) Glucose tolerance test (GTT) results are reported as the area under the curve, a quantifiable measure of glucose tolerance response at 20 weeks. (**C**) Glucose response changes over time from baseline to after STZ treatment indicating a change in glucose tolerance due to STZ treatment at 20 weeks of age in T2D. Shown as geometric means with 95% CI. (**D**) 6 h fasting glucose to identify basal hyperglycemia at 24 weeks of age. (**E**) Body weights at 24 weeks of age. Two-way ANOVA with repeated measures was used for statistical analysis, and the geometric mean with 95% CI is shown. Each symbol represents one animal (**B**,**D**,**E**). (* = *p* < 0.05, ** *p* < 0.01, *** *p* < 0.005, **** *p* < 0.001).

To gauge the effects of body weight, insulin resistance, and hyperglycemia on the immunological response to SARS-CoV-2 vaccination, we tested three commonly used diabetic mouse models [18–20]: chemo-induced T1D, diet-induced obesity, and a combination of chemo- and diet-induced T2D. Two cohorts of six-week-old C57BL/6 mice were given either a high-fat diet (HFD) (60% fat kcal) or the control diet (16% fat kcal) ad libitum for ten weeks. The high-fat and control diet mice were further divided into two additional cohorts in which half of the mice in each cohort were given a low dose of STZ (40 mg/kg, adjusted for the weight of individual mice) once a day for five days (Figure 2A). At 20 weeks of age, glucose tolerance in mice given HFD + STZ—the T2D model—was reduced when compared to mice given HFD only—the OB model (Figure 2B,C). The mice given a control diet (SCD) + STZ—the T1D model—did not have significantly reduced glucose tolerance compared to mice given the control diet only (Figure 2B,C). Following immunization and before tissue collection, at 24 weeks of age, T1D mice showed higher 6 h fasting glucose than control mice, and in T2D mice, higher fasting glucose than in OB mice was observed (Figure 2D). T1D mice showed lower body weights than control mice, and T2D mice also featured lower body weights than OB mice (Figure 2E).

Fasting blood glucose is significantly increased at 24 weeks in T1D and T2D models, while it was not increased in OB mice, suggesting that basal hyperglycemia is not present in OB mice. Still, basal hyperglycemia is observed in the T2D model, indicating hyperglycemia may be due to beta cell dysfunction and not obesity, further implying diabetes induction in the T1D and T2D mice and meeting the criteria for T1D and T2D in mice [18].

### 3.3. Cell-Mediated Immune Responses After Immunization with Adjuvanted Subunit and mRNA Vaccines in Mice with Altered Metabolic States

**Figure 3.**
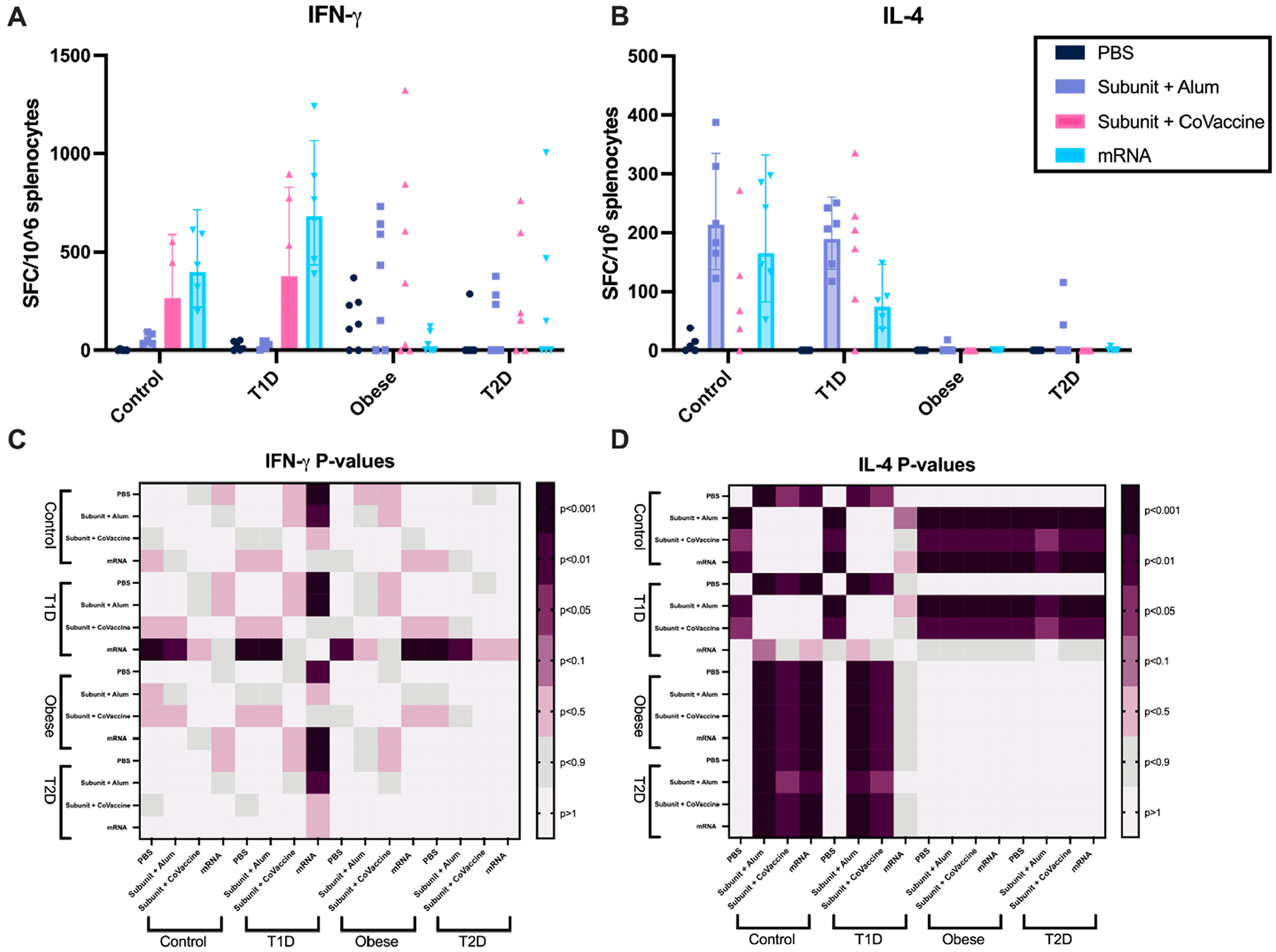
Cell-mediated immunity differs in altered metabolic states after immunization with adjuvanted subunit and mRNA vaccines. Splenocyte IL-4 and IFN-*γ* secretion was affected by metabolic changes and vaccine platform. Post-vaccine dose 2 (3 μg per dose subunit vaccine (adjuvanted with CoVaccine HT or alum) or 3 μg/mL mRNA vaccine) ELISpot responses of IFN-*γ* (**A**) and IL-4 (**B**) after stimulation with SARS-CoV-2 Spike antigen for 24 h. The range for media controls was 0–1228 SFC/well. Statistical significance between metabolic states and vaccine platform IFN-*γ* (**C**) and IL-4 (**D**) responses were reported using heatmaps, where the darker the color, the lower the p-value. A repeated-measures two-way analysis of variance (ANOVA) was used to analyze statistical differences between vaccine platforms and metabolic states for IFN-*γ* (**A**,**C**) and IL-4 (**B**,**D**). Each symbol represents one animal (**A**,**B**). Geometric mean with 95% CI is shown.

Potential variations in cell-mediated immunological responses triggered by CoVaccine HT or alum-adjuvanted protein subunit vaccines and mRNA vaccines under different metabolic states [28] were investigated. Ten days after the second immunization (day 31), splenocytes were isolated and stimulated with SARS-CoV-2 S-overlapping-peptides. In the T1D mouse model, the mRNA vaccine induced more IFN-*γ*-secreting splenocytes than the subunit vaccine adjuvanted with alum (Figure 3A). Interestingly, the alum-adjuvanted vaccine induced more IFN-*γ*-secreting splenocytes in the OB model compared to its effect in the T1D model (Figure 3A). Moreover, the mRNA vaccine induced fewer IFN-*γ*-secreting splenocytes in the OB model compared to its performance in the T1D (Figure 3A). There were no significant differences in the substantial number of IFN-*γ*-secreting splenocytes induced by the CoVaccine HT-adjuvanted vaccine between the control, T1D, T2D, and OB models (Figure 3A), suggesting that an adjuvant may induce a more T_h_1 balanced immunity regardless of metabolic state. In contrast, ELISpot analysis demonstrated that T2D and OB mice show significantly fewer IL-4-secreting splenocytes than T1D and control mice regardless of vaccine platform (Figure 3B).

### 3.4. Anti-Spike IgG Concentration After Immunization with Adjuvanted Subunit and mRNA Vaccines Is Reduced in Mice with Altered Metabolic States

**Figure 4.**
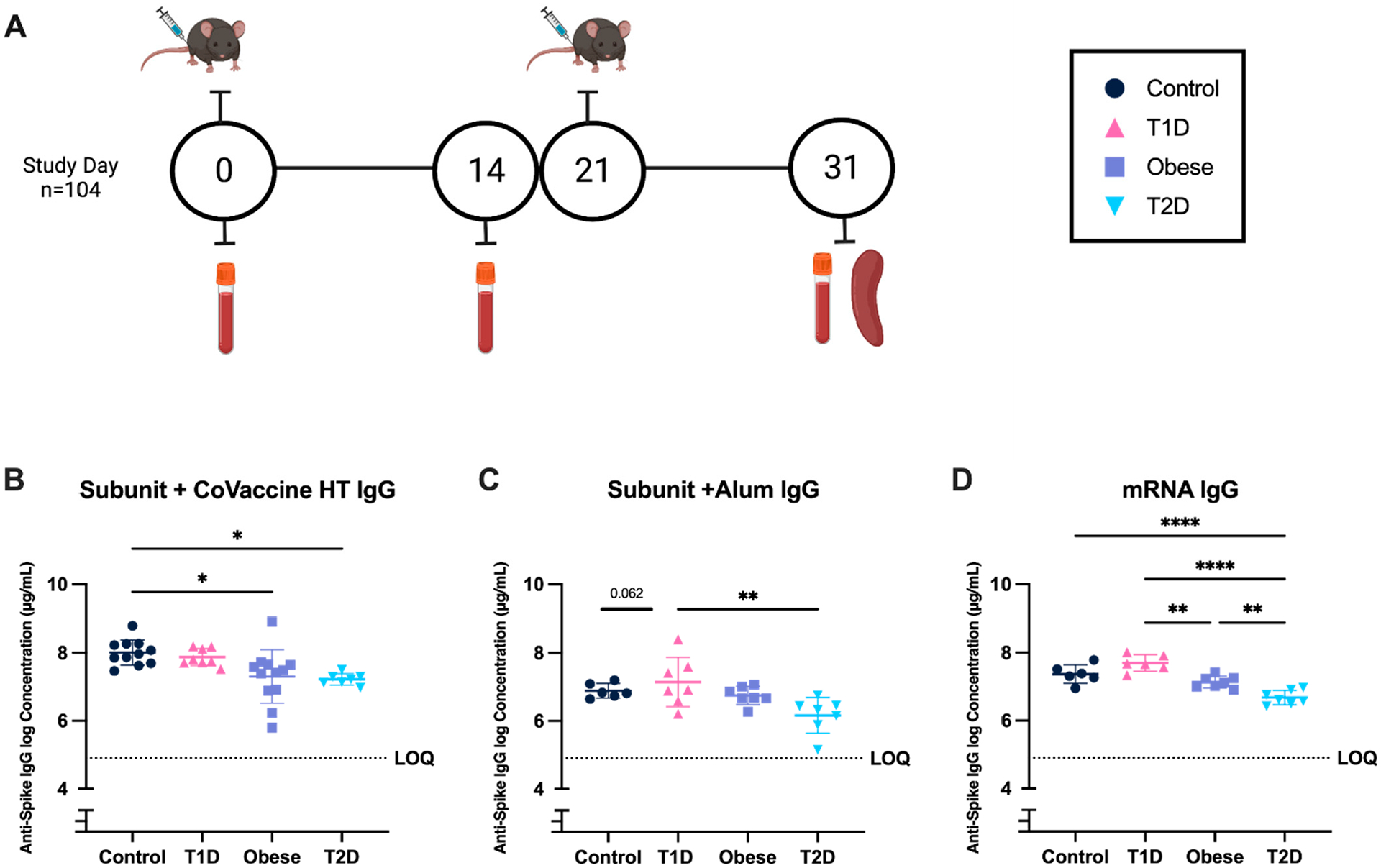
Anti-Spike IgG responses after immunization in mice with altered metabolic states. Vaccine schedule and serum collection (**A**). Baseline serum was collected prior to day zero immunization with 3 μg subunit vaccine (adjuvanted with CoVaccine HT or alum) or 3 μg mRNA vaccine (0 days). Two weeks post-dose one (14 days), serum was collected. At week three (21 days), the second immunization was given. Ten days following the second immunization (31 days), serum and spleens were collected. Anti-Spike IgG concentrations after two immunizations for subunit + CoVaccine HT (**B**), Subunit + Alum (**C**), and mRNA (**D**). A repeated-measures two-way analysis of variance (ANOVA) was used to analyze statistical differences between vaccine platforms and metabolic states within subclass and subtype responses. Each symbol represents one animal. Mean +/− SEM is shown. (* = *p* < 0.05, ** *p* < 0.01, **** *p* < 0.001).

Detection of SARS-CoV-2 spike-specific IgG in mouse serum was used to quantify antibody responses to initial immunization and ten days following the second dose (day 31) (Figure 4A). T2D mice showed reduced IgG concentrations to the full-length spike for all vaccine platforms after two doses compared to control mice (CoVaccine HT: 6.68 μg/mL ± SD 0.21 (T2D), 7.37 μg/mL ± SD 0.28 (Control), *p* < 0.05 (Figure 4B); Alum: 7.22 μg/mL ± SD 0.17 (T2D), 8.07 μg/mL ± SD 0.40 (Control) *p* = 0.06 (Figure 4C); mRNA: 6.16 μg/mL ± SD 0.52 (T2D), 6.89 μg/mL ± SD 0.21 (Control) *p* > 0.0001) (Figure 4D). After two doses, in T1D mice, Subunit + CoVaccine HT elicited an equivalent response to the mRNA vaccine, with reduced efficacy in Subunit + Alum (Figure 4B,D) mice, while the OB mice receiving Subunit + CoVaccine HT elicited lower humoral responses compared to healthy controls. When comparing vaccine platforms in T2D mice, the humoral response after two doses was significantly higher in the subunit + CoVaccine HT compared to the mRNA vaccine (*p* = 0.0007, R^2^= 0.8730).

### 3.5. Subunit Adjuvanted with CoVaccine HT and mRNA Vaccines Elicit a More Balanced Immune Response Compared to the Subunit Vaccine Adjuvanted with Alum

**Figure 5.**
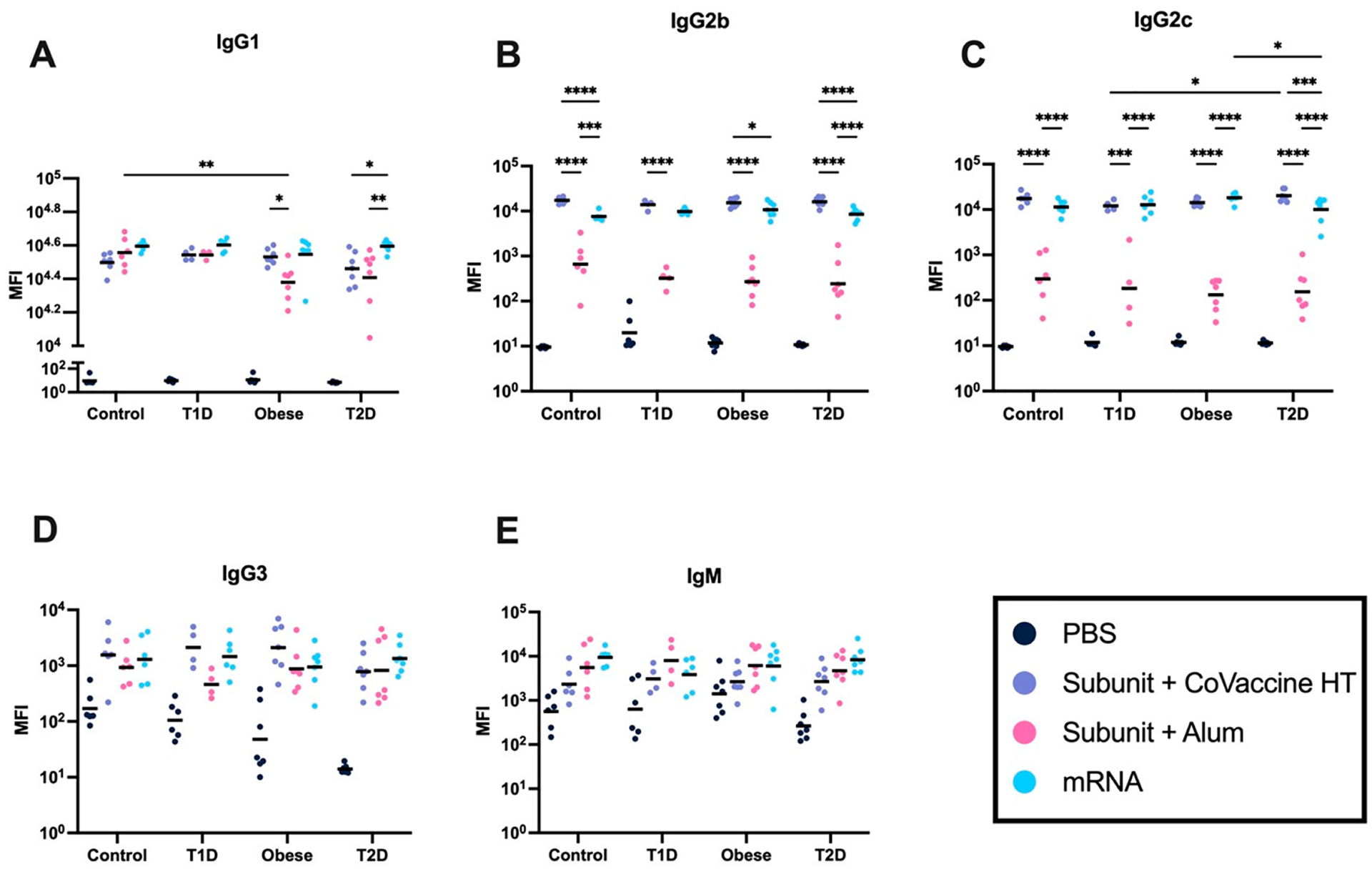

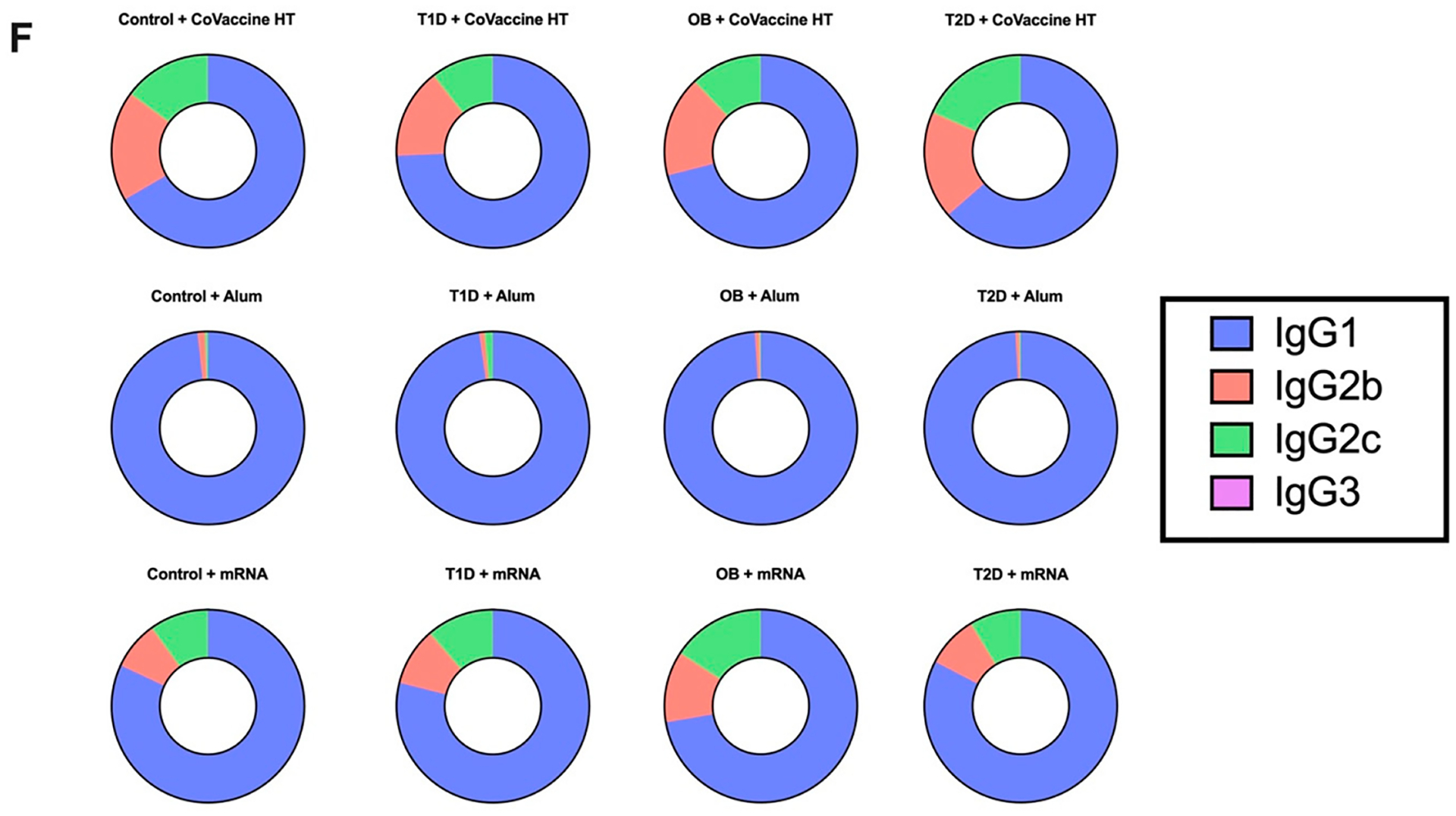
IgG subclass responses to vaccine platforms differ in mice with altered metabolic states. IgG subclass responses (MFI is reported) after two vaccine doses (3 μg subunit vaccine (adjuvanted with CoVaccine HT or alum) or 3 μg dose mRNA vaccine). IgG1 (**A**), IgG2b (**B**), IgG2c (**C**), and IgG3 (**D**). IgM isotype responses (MFI is reported) after two vaccine doses (**E**). IgG subclass ratios of total IgG, where each subclass is reported as a portion of total IgG to identify balance differences in response (**F**). A repeated-measures two-way analysis of variance (ANOVA) was used to analyze statistical differences between vaccine platforms and metabolic state within subclass and subtype responses. Each symbol represents one animal, and the black line represents the mean. (* = *p* < 0.05, ** *p* < 0.01, *** *p* < 0.005, **** *p* < 0.001).

IgG subclass levels of the antibody response to vaccination in mice with altered metabolic states were evaluated. IgG responses in the case of T2D are strongly IgG1 biased compared to other metabolic states after immunization with the alum and mRNA vaccine platforms (Figure 5A). Spike subunit vaccine adjuvanted with Alum elicited lower IgG2b responses than other platforms (Figure 5B). An additional IgG2 murine subclass, IgG2c, shows similar trends, and an increase in IgG2c is seen in the T2D mouse model (Figure 5C). mRNA also elicits a relatively higher IgG2c response in OB mice than alum- and CoVaccine HT-adjuvanted subunit vaccines (Figure 5C). IgG2a levels were not assessed, as this mouse model does not typically elicit IgG2a [47]. A subclass associated with the induction of effector functions [48], IgG3, is elicited uniformly across all the metabolic states and between vaccine platforms (Figure 5D). IgM responses were also assessed, and the results showed uniform levels elicited across all metabolic states, with no statistical differences observed between each group’s median fluorescence intensity (MFIs) (Figure 5E).

Overall differences are seen between vaccine platforms; however, limited alterations of responses are seen between metabolic states (Figure 5F). Compared to alum-adjuvanted and mRNA vaccines, a more balanced IgG response across all metabolic states is seen in the CoVaccine HT-adjuvanted subunit vaccine (Figure 5F).

### 3.6. mRNA Vaccines Elicit Consistently High Virus-Neutralizing Antibody Responses Under All Metabolic Conditions

**Figure 6.**
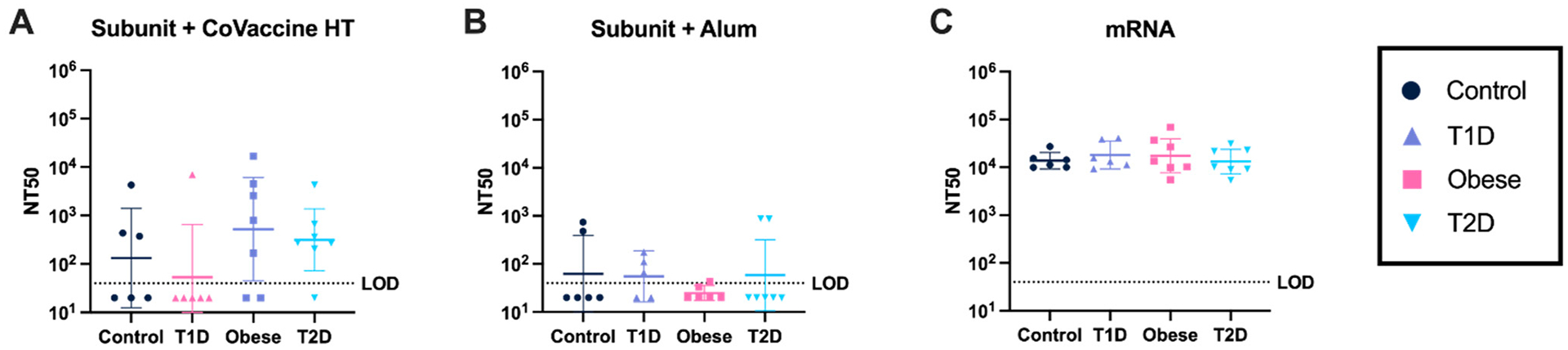
Virus neutralization responses after two vaccine doses. NT50 (50% neutralizing titer) was measured using a microneutralization assay after two vaccine doses of Subunit + CoVaccine HT (3 μg) **(A)**, Subunit + Alum (3 μg) **(B)**, and mRNA (3 μg) **(C)** at day 31. Each symbol represents one animal, geometric mean with 95% CI is shown. The horizontal dotted line represents the limit of detection (LOD). Two-way ANOVA with repeated measures was used for statistical analysis of neutralizing antibody responses between metabolic state and vaccine platform.

Virus-neutralizing antibody titers were determined after two doses of each vaccine. Neutralizing responses were varied among the adjuvanted subunit vaccines in all metabolic states (Figure 6A), while subunit adjuvanted with alum responses show limited neutralizing titers in all metabolic groups (Figure 6B). Mice receiving the mRNA vaccine showed high neutralizing titers (Figure 6C).

### 3.7. Adjuvanted Subunit Vaccines Elicit Antibodies with Stronger Avidity After Ten Days Post-Second Immunization Compared to mRNA Vaccines

**Figure 7.**
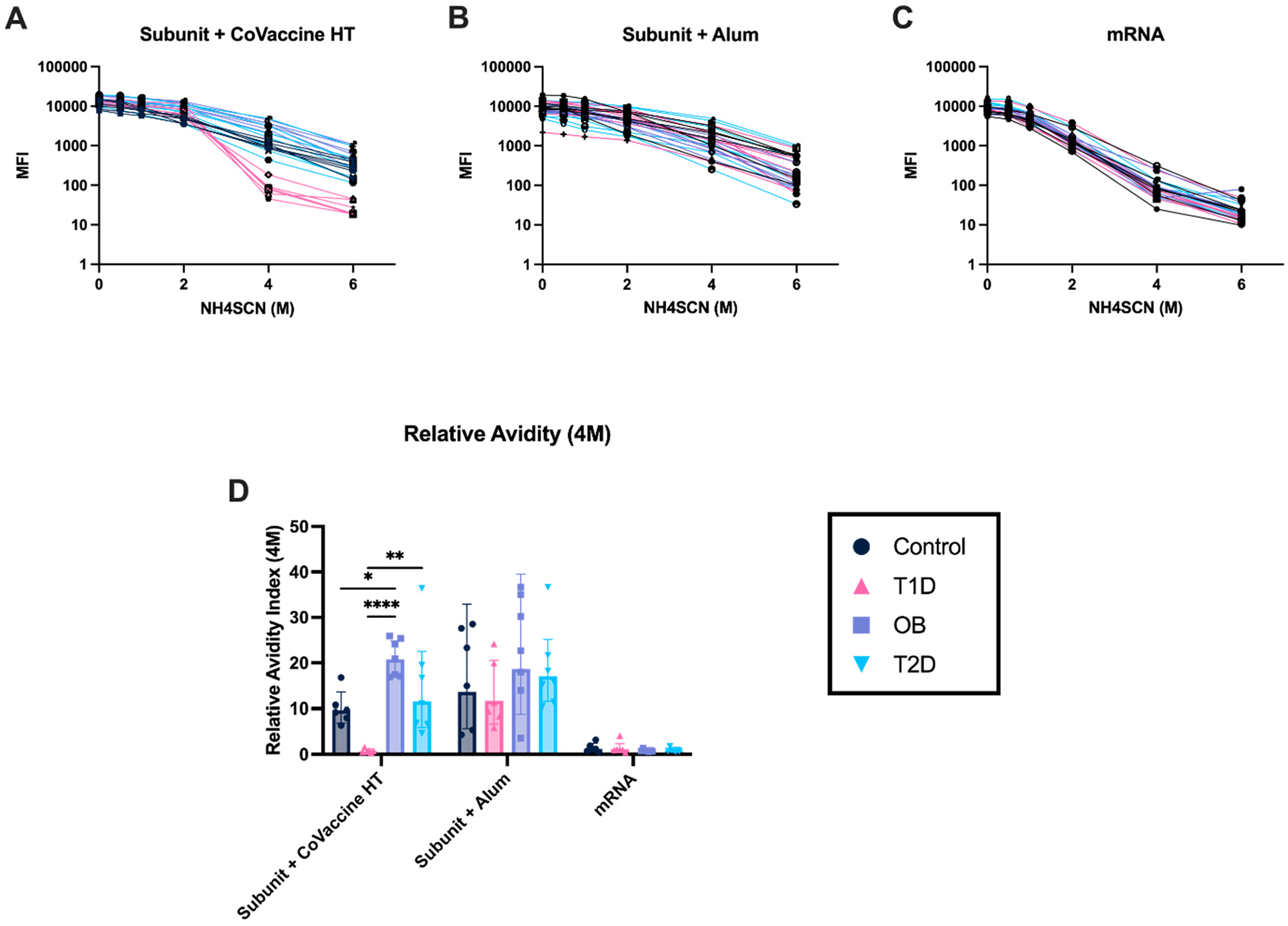
Avidity profiles induced by various vaccines in altered metabolic states. Antibody avidity observed after two vaccine doses of Subunit + CoVaccine HT (3 μg) **(A)**, Subunit + Alum (3 μg) **(B)**, and mRNA (3 μg) **(C)**. Relative avidity at 4 M NH_4_SCN was measured as the percentage of original antibody remaining bound to antigen after chaotropic treatment **(D)**. Each line represents one animal **(A–C)**, and each symbol represents one animal, where the bar represents the mean **(D)**. Geometric mean with 95% CI is shown. Statistical analysis of relative avidity was completed using a two-way ANOVA with repeated measures. (* = *p* < 0.05, ** *p* < 0.01, **** *p* < 0.001)

Antibody avidity against the SARS-CoV-2 spike protein was measured as the percentage of antibodies eluted from bound antigens in a solid phase assay by the chaotropic (dissociating) agent, sodium thiocyanate (NH_4_SCN) [49,50]. The results showed significant differences between metabolic groups in mice immunized with the Subunit + CoVaccine HT vaccine. In the CoVaccine HT-adjuvanted group, T1D mice had significantly lower relative avidity than the three other metabolic groups (Figure 7A,D). Furthermore, in the CoVaccine HT group, the OB mice had higher relative avidity than the control and T2D groups (Figure 7D). The same trend held in the alum group, but no significant differences in results were observed. In both adjuvanted subunit vaccine groups, the OB mice showed no significant differences between metabolic states observed (Figure 7A,B,D). Additionally, mice immunized with the mRNA vaccine had much lower relative avidity than both subunit vaccine groups (Figure 7C,D).

## 4. Discussion

The role of vaccination in global health is indisputable, as it effectively curtails disease burden, reduces morbidity and mortality rates, and contributes significantly to the reduction of health inequities, making it an essential approach in controlling and preventing various diseases globally. With the rise in health disparities during the COVID-19 pandemic, especially in those with comorbidities [51–53], immunizing and protecting at-risk populations became imperative. A lack of research surrounding vaccine efficacy in those with altered metabolic states hinders the development of public health interventions. Previous literature has shown reduced vaccine efficacy for influenza vaccines in individuals with altered metabolic states [54]. In a recent study, those with and without T1D had robust humoral responses to vaccination [55]. Those with T2D had reduced anti-Spike IgG titers compared to healthy controls after immunization with the BNT162b2 mRNA COVID-19 vaccine [55]; however, analysis of the quality of these antibodies remains unaddressed.

Comparison of vaccine responses to different vaccine platforms in healthy control states allows for optimizing doses for further investigation into responses elicited within populations with altered metabolic states. Previous studies have shown that optimized and effective vaccine doses of subunit adjuvanted with CoVaccine HT elicited strong humoral immunity in mice [28,29] and non-human primates [46,56–58]; however, previous research only enlisted healthy mice. Our novel study uses mouse models of diabetes and obesity to identify differences between healthy mice and altered metabolic states regarding immune responses to SARS-CoV-2 vaccination.

Elevated T_h_1 and T_h_17 markers and depressed T_h_2 markers have been seen in those with obesity and T2D [22,23]. While T_h_1 is generally associated with a robust antiviral response, obesity and DM are associated with poor outcomes of COVID-19. Therefore, a vaccine with an adjuvant that consistently stimulates the highest T_h_1/T_h_17 versus T_h_2 responses may initiate robust protection in OB and DM models. Furthermore, those with obesity and DM may require elevated IgG levels and a more focused immune profile (potentially geared more towards a T_h_1 response) for protection. Few studies have analyzed vaccine-induced cellular immunity in OB and DM models, and observational studies on vaccine effectiveness remain biased due to confounders (e.g., depletion of susceptibles [59]). Animal models, therefore, are an essential starting point for studying the biological effects of obesity and DM on vaccine responses. This study provides some insight into immune profiles associated with altered metabolic states post-SARS-CoV-2 immunization.

The three distinct models of obesity, T1D, and T2D, used herein, provide a comprehensive outline of immunological responses and vaccine efficacy in animals with metabolic disturbances. The induction of obesity was successful through the application of a high-fat diet. The strain of mice used can determine the susceptibility to diet-induced metabolic changes. T_h_1-biased C57BL/6 male mice are one of the most sensitive mouse strains to diet-induced obesity (DIO) and are used throughout diabetes research [18]. Outbred strains of mice more closely match human genetic diversity; however, they are less susceptible to DIO [60]. Diabetes was induced using the chemotactic agent STZ, which ablates pancreatic beta islet cells and reduces glucose tolerance. Chemotherapeutic actions of STZ have been shown to suppress naïve immune activity levels; however, the effects of STZ on the immune system are likely due to systemic activity rather than direct effects on the immune cells themselves. This is partly due to STZ entering the cell through the GLUT2 transporter, while immune cells rely on GLUT1 for glucose transport [61].

Diabetes mellitus is associated with increased production of IFN-*γ* by T cells prior to immunization [22]. We found that the frequency of antigen-specific IFN-*γ* responses in mRNA-immunized mice was reduced in OB mice compared to alternative vaccine platforms. A reduction in IFN-*γ* driven cellular immune responses decreases vaccine-induced protection against SARS-CoV-2 [62], inferring obesity reduces mRNA vaccine-induced protection. Exacerbated inflammation of obese adipose tissue affects the pancreatic β cells, causing hyperinsulinemia, leading pro-inflammatory cytokines to induce β-cell apoptosis. These combined mechanisms can produce advanced glycation and T2D [63,64]. The unique immune environment of adipose tissue is impacted by the tissue mass expansion and lipolysis activation, which occurs after diet-induced obesity [65], and is likely the cause of cell-mediated impaction in these mice. Further investigations exploring mechanistic insights into the antibody responses, including analysis of THF (IL-21) and TFR (IL-10) would be beneficial next steps to understanding the strength of vaccine responses to the different vaccine platforms.

This investigation into the impact of altered metabolic states on the immune response to immunization with adjuvanted subunit and mRNA vaccines in mice reveals a note-worthy reduction in the concentration of anti-spike IgG compared to control (normal) mice. These findings highlight the implications of metabolic conditions on vaccine efficacy and warrant further exploration to optimize immunization strategies for individuals with altered metabolic profiles [28]. In the context of vaccination, a reduction in anti-spike IgG levels suggests that the vaccine’s ability to induce a robust immune response has been compromised. This could have implications for the overall efficacy or durability of the vaccine to confer protection against SARS-CoV-2. Furthermore, the observation of reduced anti-spike IgG levels signal the need for enhanced vaccination strategies to improve immune responses in at-risk populations. Further research is encouraged to understand the underlying mechanisms and factors influencing the humoral immune response.

Elicited diverse IgG subclasses provide insight into directed B-cell responses and the priming of immune responses to future viral infection [48]. Subunit vaccines adjuvanted with CoVaccine HT and mRNA vaccines stimulate a more balanced immune response than the more T_h_2-skewed response from the subunit vaccine adjuvanted with Alum, characterized based on increased IL-4 and decreased IFN-γ secreting splenocytes compared to the other platforms. Further evidence is given by increased IgG1 titers relative to IgG2b and IgG2c compared to the other platforms. However, the CoVaccine HT adjuvanted subunit vaccine elicited higher levels of IgG2c in T2D compared to T1D, and the mRNA vaccine showed decreased IgG2c responses in T2D compared to OB, possibly associated with a T_h_1-biased proinflammatory state in obesity [14,66]. Thus, the subclass responses observed are consistent with T_h_1 skewing, and the results support T_h_1-biased responses in C57BL/6 mouse models of diabetes [67].

These findings suggest that CoVaccine HT-adjuvanted subunit and mRNA vaccines may offer advantages in inducing a more balanced immune response. These results have significant implications for vaccine design and development, as they highlight the potential of mRNA and CoVaccine HT-adjuvanted subunit vaccines in achieving robust and comprehensive protection against SARS-CoV-2. Moreover, the observed differences in IgG2c levels between T2D and T1D, as well as between T2D and OB, suggest that metabolic states may influence the immune responses to these vaccines. The general process of B cell activation, antibody production, and subsequent immune functions are central to the success of vaccinations in providing protection against SARS-CoV-2 [16,17], and reduced IgG2c responses may indicate effects in vaccine efficacy due to altered metabolic states. Further research in diverse human populations and exploring the underlying mechanisms responsible for these immune responses will be crucial in tailoring vaccination strategies to specific comorbidities and optimizing vaccine efficacy.

Responsible for regulating and tolerating the immune response and acting as a first line of defense [68], IgM responses were uniform and not skewed due to alterations in the metabolic state, indicating initial humoral immune responses due to vaccination are not impaired by diabetic state. Differences elicited between vaccine platforms are likely due to different response kinetics to each vaccine and likely result from differences in the introduction of antigenic components. While subunit vaccines directly introduce the antigenic components, mRNA vaccines involve the active synthesis of proteins within the vaccinated cells [69,70]. It is important to note that while the initial protein production starts relatively quickly for mRNA vaccines, the immune response takes time to develop and mature.

Not only is the quantity of antibodies important in eliciting desired responses to vaccination, but the quality of these antibodies is essential in generating protective responses. As a measure of partial protection [16,17], neutralizing antibodies constitute one measure of vaccine functionality in in-vivo studies of SARS-CoV-2. However, previously there has been a gap in understanding the formation of high-quality neutralizing responses in individuals with altered metabolic states after vaccination, such as those with T1D, T2D, and obesity.

Our data suggest there are variations in neutralization responses to the original strain of SARS-CoV-2 among different subunit vaccines in all metabolic states. However, the mice receiving the mRNA vaccine exhibit a strong specific neutralizing response, with neutralizing titers significantly higher than those observed for other vaccine platforms. Interestingly, neither a diabetic state nor obesity seems to influence the neutralizing titers elicited by the mRNA vaccine, indicating consistent and robust responses in these metabolic conditions. This reinforces previous studies indicating modified mRNA vaccines, such as the one used in this study, induce robust and potent neutralizing antibody responses through T follicular helper (Tfh) cell and germinal center (GC) initiation, thereby fostering immunoglobulin class switch, affinity maturation, and long-term B cell memory [71–74].

On the other hand, trends suggest that obese mice (both OB and T2D mice) tend to have higher neutralizing titers compared to healthy control mice and T1D mice after receiving the subunit vaccine adjuvanted with CoVaccine HT. Considering the importance of broad neutralizing responses to confer cross-reactive protection against SARS-CoV-2 variants and subvariants, further investigations are warranted to identify differences in the neutralization profiles elicited by each vaccine platform. Furthermore, antigen-specific Th1 T-cell response is reduced in diabetic models, which may contribute to poor outcomes in people with diabetes. Understanding the interplay between altered metabolic states and vaccine-induced neutralizing responses is crucial for optimizing vaccine efficacy and ensuring comprehensive protection against SARS-CoV-2, including its variants.

Virus-specific neutralizing responses are essential for acute events, including out-breaks; however, high-quality and broadly reactive antibody responses after affinity maturation are needed for long-term and cross-reactive protection. Measures of affinity maturation, including avidity, provide evidence of the quality of vaccine responses determined by the binding strength of an antibody population to a complex antigen [49,75]. Reduced avidity observed in specific vaccine platforms or metabolic states may indicate impaired affinity maturation caused by chronic inflammation and altered Tfh activity.

Diverse antibody responses are seen, especially in the subunit vaccine adjuvanted with CoVaccine HT, with high anti-spike IgG specific concentrations and increased relative avidity. Diabetic states impaired avidity to a greater extent relative to obesity, and T1D impaired avidity more than T2D in all vaccine platforms. These findings indicate avidity changes are associated with physiological differences between diabetes and obesity as well as *β* cell ablation effects in the pancreas. Further investigation is needed to identify the causality of B cell maturation differences over time in these vaccine platforms; however, three weeks post-vaccination is likely not enough time to stimulate complete evolution of affinity maturation in mRNA vaccines [76].

Waning protection over time, suboptimal vaccine efficacy, and immune response can lead to future breakthrough infections. Adjuvanted vaccines likely promote robust affinity maturation due to prolonged antigen exposure’s “depot effect” [77,78], while modified mRNA vaccines promote strong affinity maturation through Tfh cell and GC activation [71–74]. Reduced avidity observed in certain vaccine platforms or metabolic states may indicate impaired affinity maturation caused by chronic inflammation and altered Tfh activity. These differences may be essential when considering vaccinating populations affected by metabolic syndromes. Further investigation is needed to elucidate the physiological mechanisms that lead to differences in antibody avidity between metabolic syndromes. More specifically, investigations into the patterns of LNP-delivered self-amplifying RNA vaccines and long-term exposure to the antigen mediation of this process would be of interest. Furthermore, it would be interesting to learn whether a heterologous prime–boost strategy with an mRNA LNP prime and a subunit CoVaccine HT boost could further produce higher and more consistent neutralizing Ab titers.

This study provides valuable, novel insights into the impact of altered metabolic states, specifically obesity and diabetes, on the immune responses elicited by SARS-CoV-2 vaccines. Our findings show that T2D and OB mouse models exhibit reduced humoral and cell-mediated immune responses following vaccination compared to healthy mice. The observed decrease in immune responses among diabetic and OB mice suggests that chronic inflammatory states associated with these metabolic disorders may affect vaccine immunogenicity.

These findings are consistent with previous studies highlighting the immunomodulatory effects of obesity and diabetes on the immune system [30–32]. Notably, our study demonstrates that mRNA vaccines maintain strong neutralizing responses across all metabolic states, indicating their potential as an effective vaccine platform even in the context of altered metabolic states. On the other hand, adjuvanted subunit vaccines show decreased humoral and cell-mediated immune responses in T2D mice, while providing increased antibody avidity in diabetic and OB mice compared to healthy mice. These results indicate the importance of considering vaccine platforms and their specific immune mechanisms when designing vaccines for populations with altered metabolic states or other metabolic disorders.

Understanding the mechanisms underlying the reduced immune responses in altered metabolic states is important for developing strategies to enhance vaccine efficacy. Addressing chronic inflammation and metabolic dysregulation associated with obesity and diabetes may be essential in improving the immunogenicity and durability of SARS-CoV-2 vaccines. Furthermore, our findings emphasize the need to investigate optimized vaccines tailored to the unique immune characteristics of individuals with obesity and diabetes. This may involve exploring alternative vaccine platforms, formulation modifications, or the inclusion of specific adjuvants to increase immune responses in these populations, especially given that over half a billion people are living with DM globally [79].

Novel to the scientific community, this study analyzed the effect of hyperglycemia alone (T1D), hyperglycemia with insulin resistance (T2D), and increased body weight without necessarily being diabetic (OB). Our investigation highlights the challenges posed by altered metabolic states on vaccine-induced immune responses against SARS-CoV-2 and potentially other diseases. It emphasizes the need for tailored vaccination strategies in at-risk populations, and addressing these challenges will be vital in ensuring widespread and durable protection against COVID-19 and other infections, such as influenza. Continued research in this field will pave the way for more informed and precise approaches to combat infectious diseases, ensuring global health security and resilience in the face of emerging health challenges.

## Data Availability

The data presented in this study are available on request from the corresponding author.
